# Detection of New H5N1 High Pathogenicity Avian Influenza Viruses in Winter 2021–2022 in the Far East, Which Are Genetically Close to Those in Europe

**DOI:** 10.3390/v14102168

**Published:** 2022-09-30

**Authors:** Norikazu Isoda, Manabu Onuma, Takahiro Hiono, Ivan Sobolev, Hew Yik Lim, Kei Nabeshima, Hisako Honjyo, Misako Yokoyama, Alexander Shestopalov, Yoshihiro Sakoda

**Affiliations:** 1 Laboratory of Microbiology, Faculty of Veterinary Medicine, Hokkaido University, Kita 18, Nishi 9, Kita-ku, Sapporo 060-0818, Hokkaido, Japan; 2 International Collaboration Unit, International Institute for Zoonosis Control, Hokkaido University, Kita 20, Nishi 11, Kita-ku, Sapporo 001-0020, Hokkaido, Japan; 3 Ecological Risk Assessment and Control Section for Environmental Biology and Ecosystem, Biology Division, National Institute for Environmental Studies, Onogawa 16-2, Tsukuba 305-8506, Ibaraki, Japan; 4 Institute of Virology of the Federal Research Center of Fundamental and Translational Medicine, Novosibirsk State University, Bild 2, Timakova St., Novosibirsk 630117, Russia

**Keywords:** bird migration, genetic profile, high pathogenicity avian influenza, H5N1, wild birds

## Abstract

Many high pathogenicity avian influenza (HPAI) cases in wild birds due to H5N1 HPAI virus (HPAIV) infection were reported in northern Japan in the winter of 2021–2022. To investigate the epidemiology of HPAIVs brought to Japan from surrounding areas, a genetic analysis of H5 HPAIVs isolated in northern Japan was performed, and the pathogenicity of the HPAIV in chickens was assessed by experimental infection. Based on the genetic analysis of the hemagglutinin gene, pathogenic viruses detected in northern Japan as well as one in Sakhalin, the eastern part of Russia, were classified into the same subgroup as viruses prevalent in Europe in the same season but distinct from those circulating in Asia in winter 2020–2021. High identities of all eight segment sequences of A/crow/Hokkaido/0103B065/2022 (H5N1) (Crow/Hok), the representative isolates in northern Japan in 2022, to European isolates in the same season could also certify the unlikeliness of causing gene reassortment between H5 HPAIVs and viruses locally circulating in Asia. According to intranasal challenge results in six-week-old chickens, 50% of the chicken-lethal dose of Crow/Hok was calculated as 10^4.5^ times of the 50% egg-infectious dose. These results demonstrated that the currently prevalent H5 HPAIVs could spread widely from certain origins throughout the Eurasian continent, including Europe and the Far East, and implied a possibility that contagious viruses are gathered in lakes in the northern territory via bird migration. Active monitoring of wild birds at the global level is essential to estimate the geographical source and spread dynamics of HPAIVs.

## 1. Introduction

As in the last outbreaks of high pathogenicity avian influenza (HPAI) in the Far East, many H5N8 HPAI virus (HPAIV) infections in poultry and wild birds were reported in winter 2020–2021, following the continuous notifications of HPAIV infections in Europe since autumn 2020. The origin of almost all H5 HPAIVs recently circulating worldwide was considered an isolate in Guangdong in 1996 and evolved to ten genetically distinct virus clades with many subclades [1]. Among those, H5 HPAIV clade 2.3.4.4 was identified and rapidly spread in winter 2014–2015 and is currently the most prevalent worldwide [2]. Genetic analysis revealed that not only dominant strains in Europe in winter 2019–2020 but also those in winter 2020–2021 belong to clade 2.3.4.4b and have been introduced to Japan and the Republic of Korea, possibly by bird migration [3,4,5,6]. Outbreaks of H5N8 HPAIV were, in the Far East, once terminated in spring 2021 but continuously reported in European poultry and wild birds until summer 2021 [7]. Moreover, infections with H5N1 HPAIVs, which could be generated as reassortment between H5N8 HPAIVs circulating in Europe and low-pathogenicity avian influenza viruses circulating locally, have been dominant in European countries since late spring 2021, contrary to the decrease of the reported cases of H5N8 HPAIV infections [8]. It was confirmed that two types of H5 viruses with different hemagglutinin (HA) in the genetic pool were already circulating in autumn 2021; one had been continuously present with genetic drift among the European bird population since autumn 2020, and the other, which originated in African countries but could be widely spread throughout European countries [8] had been detected since October 2021. Under such a circumstance, many gene-reassorted viruses, including H5N1, were generated, and dominant H5N1 reassorted viruses were further transferred from Europe to African, North American, or Asian countries in winter 2021–2022 [9,10,11,12]. Phylogenetic analyses have already revealed that two types of H5 HPAIVs invaded southern Japan at the end of 2021. H5N1 HPAIV with slight mutations on the HA gene from the parental H5N8 HPAIV and H5N8 HPAIVs, similar to European dominant strains in winter 2020–2021, have been confirmed, whereas no H5N1 HPAIVs dominant in Europe in the same season have been detected [13].

According to the Ministry of Environment regulations, a definite diagnosis of HPAIV infections in wild birds in Japan had been virus isolation using tracheal or cloacal swab samples of birds. However, before winter 2021–2022, considering the serious damages of H5 HPAI outbreaks in winter 2020–2021 in Japan and continuous H5 HPAI cases in Europe since 2020, amino acid sequencing of the isolate was attempted using the gene amplicon directly from swab samples instead of propagated virus isolates in chicken embryos to shorten the diagnosis period by skipping the virus isolation process. An effective modification in HPAIV diagnosis in wild birds was expected to contribute directly to quick decision-making to minimize the damages of HPAIV infections.

**Figure 1:**
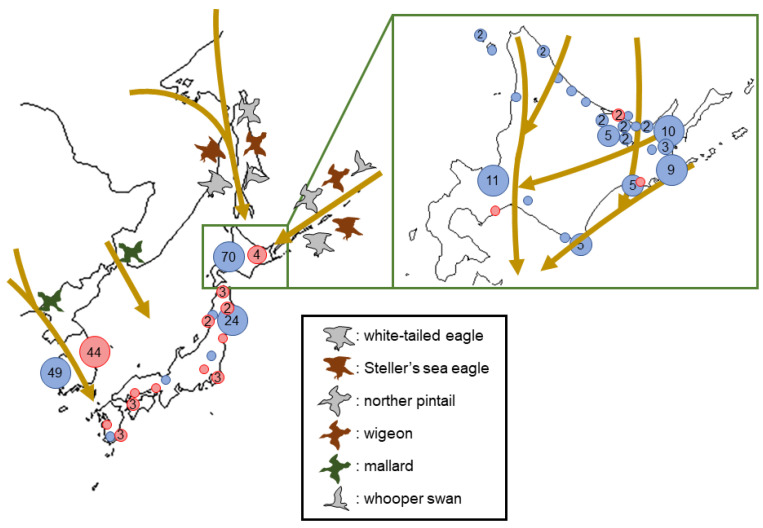
Number of high pathogenicity avian influenza (HPAI) outbreaks in poultry and cases in wild birds in the Far East between the beginning of November 2021 and the end of May 2022. Numbers in the circle indicate the reported accumulated numbers of HPAI outbreaks in poultry (red) and cases in wild birds (blue) in each area or country. A circle without a number indicates a single outbreak/case in the area. The yellow arrows indicate the general flyways of migratory birds in autumn-winter in Japan. Figures of colored structures indicate the migratory flyway of each migratory bird. Outbreak reports used in this figure were derived from the EMPRES-I + Global Animal Disease Information system (the Republic of Korea) [19], the HPAI outbreak database of the Ministry of Agriculture, Forestry and Fisheries [17], and the HPAI case database of the Ministry of Environment (Japan) [18].

There are four major bird migration routes into Japan, which seriously affect the occurrences of HPAI cases in Japan [14,15,16]. To the northern part of Japan, migratory birds fly through Sakhalin Island or Kuril Islands—Kamchatka Peninsula, originating from Siberia or the Bering Sea—Alaska. Migration of raptors including white-tailed eagle and Steller’s sea eagle, and some aquatic birds including whooper swan, wigeon and northern pintail are flying to the northern part of Japan in these pathways. Some aquatic birds including the mallard are recognized in two migratory routes from the Asian continent to Japan; either direct to the central part of Japan or via the Korean Peninsula to the southern part of Japan. During winter 2021–2022, through these migratory routes, 25 HPAI outbreaks in poultry and 98 HPAI cases in wild birds (except 9 positives isolated from environment samples) were brought to Japan and the Republic of Korea, respectively (Figure 1) [17,18]. On Hokkaido Island in particular, situated in the northern part of Japan, four outbreaks in poultry farms and seventy cases in wild birds were confirmed near the flyways of migratory birds (Figure 1). In this study, H5N1 HPAIVs detected from wild birds and poultry in Hokkaido and the northern part of Honshu Islands of Japan between January and May 2022 were genetically analyzed to investigate the epidemiology of H5 HPAIVs brought to Japan by comparing the gene sequences of the virus isolates in surrounding areas. Furthermore, the pathogenicity of a representative HPAIV strain isolated during 2021–2022 HPAIV events in chickens was experimentally assessed.

## 2. Materials and Methods

Under the new regulations of the definitive diagnosis of HPAIV infections in wild birds by the Ministry of Environment, Japan, AIV infection in swab samples was first investigated by reverse transcription loop-mediated isothermal amplification described previously [20]. Briefly, viral RNA was extracted from tracheal or cloacal swabs of sample birds using the EZ1 Virus Mini Kit version 2.0 (Qiagen, Hilden, Germany). The presence of the matrix gene of the influenza A virus was confirmed by its amplification using a Loopamp RNA Amplification Kit (Eiken Chemical, Co., Ltd., Tokyo, Japan). Viral RNAs were extracted from positive samples with the matrix gene of influenza A virus using QIAamp viral RNA mini Kit (Qiagen), and the presence of the H5 HA gene was investigated by quantitative reverse transcription-polymerase chain reaction (RT-qPCR) using AgPath-ID One-Step RT-PCR Reagents (Thermo Fisher Scientific, Waltham, MA, USA) on a StepOnePlus Real-Time PCR System as described in Heine et al. [21]. If viral matrix and H5 HA genes were confirmed, the swab samples in minimum essential medium (Nissui, Tokyo, Japan) were inoculated into the allantoic cavity of embryonated chicken eggs to isolate infectious viruses. The collected allantoic fluid was tested for the growth of influenza A viruses by the hemagglutination assay, and the isolated viruses were subtyped by hemagglutination inhibition and neuraminidase inhibition tests using chicken antisera against the referenced influenza viruses [22]. To determine the pathogenicity of the isolate in chickens, a viral sequence was performed to confirm the presence of the multiple basic amino acid residues at the HA gene. Furthermore, the full-length viral genome of all eight gene segments was performed in the representative strains selected according to the spatial and temporal distribution and the host species by either Sanger sequencing or illumina Miseq sequencing. Direct sequencing of each gene was performed using BigDye Terminator version 3.1, a Cycle Sequencing Kit (Life Technologies, Carlsbad, CA, USA), and a 3500 Genetic Analyzer (Life Technologies). For next-generation sequencing, MiSeq libraries were prepared using Next Ultra RNA Library Prep Kit and Multiplex Oligos for Illumina (New England Biolabs, Ipswich, MA, USA) and sequenced on the MiSeq using MiSeq reagent kit version 3 (Illumina, San Diego, CA, USA). Reads were de novo assembled using FluGAS version 2 (World Fusion).

The HA and neuraminidase (NA) genes of the nucleic acids in the samples from which no infectious viruses were isolated were amplified by one-step RT-PCR to perform direct sequencing as described previously [20]. The pathogenicity characteristics of the amplicon were evaluated according to the presence or absence of multiple basic amino acid residues at the cleavage site of the HA gene. NA subtypes were determined by nucleotide sequence following to RT-PCR using the specific primer for the NA gene.

The nucleotide sequence datasets from the representative H5Nx clade 2.3.4.4 strains, H1N1, H2N1, H3N1, H6N1, and H7N1, including outgroup viruses downloaded from the Global Initiative on Sharing All Influenza Data (GISAID) and the DNA Data Bank of Japan (DDBJ), were prepared with adequate alignment for creating phylogenetic trees. The maximum-likelihood method under 1000 replication was applied to construct the phylogenetic tree of each gene segment using the best-fit general time-reversible model of the nucleotide substitution with gamma-distribution rate variation among sites (with four rate categories, Γ) in MEGA 7 [23].

An H5N1 HPAIV isolate, A/crow/Hokkaido/HU-1/2022 (H5N1) was isolated from a dead crow collected by us in Sapporo, Hokkaido in May 2022, and genetically investigated to determine the nucleotide sequence of all the eight viral gene segments. For one H5N1 HPAIV isolate, A/wigeon/Sakhalin/37M/2021 (H5N1), the infectious virus was isolated from embryonated chicken eggs through the active survey in Sakhalin, the eastern part of Russia, in September 2021 and genetically investigated to determine the nucleotide sequence of the viral HA and NA genes. In addition, tissue and swab samples of the chicken or emu carcasses found at the H5N1 HPAIV positive farms were kindly shared from the livestock hygiene services of Hokkaido Prefecture for further genetic investigation. Lung homogenates of poultry (chicken and emu) were also inoculated into the allantoic cavity of embryonated chicken eggs to isolate the infectious viruses to determine the nucleotide sequence of all the eight viral gene segments. Direct sequence was conducted using the extracted viral RNA from the tissue samples of the poultry carcasses if no infectious viruses were isolated. All the gene sequence data obtained in this study were submitted to GISAID or DDBJ, and the accession numbers of registered virus gene data were described in Supplemental Table S1.

To assess the pathogenicity of H5N1 HPAIV in chickens, the survival rates of the chickens intranasally challenged with an HPAIV with a variety of infectivity were calculated. A/crow/Hokkaido/0103B065/2022 (H5N1) (Crow/Hok), isolated from a dead crow found in an urban garden in the capital of Hokkaido Prefecture, was diluted 10^2.0^, 10^3.0^, 10^4.0^, 10^5.0^ and 10^6.0^ times of 50% egg infectious dose (EID_50_)/0.1 mL with phosphate-buffered saline. One hundred microliters of each diluted virus were intranasally inoculated into each of four 6-week-old chickens hatched from embryonated chicken eggs and fed at Hokkaido University. All chickens were daily monitored on clinical manifestations and survival for 14 days post virus challenge (dpc). The 50% chicken lethal dose (CLD_50_) of Crow/Hok was introduced by the Reed and Munch method [24]. All animal experiments were carried out at the animal BSL-3 facility at the Faculty of Veterinary Medicine, Hokkaido University, which has been accredited by the Association for Assessment and Accreditation of Laboratory Animal Care International since 2007, with approval from the Institutional Animal Care and Use Committee of the Faculty of Veterinary Medicine, Hokkaido University (approval number: 18-0037).

## 3. Results

In the winter of 2021–2022, a total of 76 migratory or locally resident birds were reported to be initially positive for AIV antigen detection or gene detection. Of these, 72 birds were officially diagnosed as H5 HPAIV positive either by virus isolation or detection. Twenty-six out of 29 raptors which were positive for AIV gene detection, were officially diagnosed as H5 HPAIV infection. One carcass of a crow with H5 HPAIV positive was collected by our own survey in Hokkaido. Among these positive samples, a total of 29 virus stains isolated or detected from wild birds in Japan were used for the genetical analysis (Supplemental Table S1). Of these, 16 viruses were performed for a full-genome sequence of all eight gene segments. Direct sequencing was performed in 13 samples negative for virus isolation but positive for the matrix and H5 HA genes. All 16 isolated and 7 out of 13 gene-detected viruses were subtyped as H5N1. However, the other 6 gene-detected viruses were subtyped only as H5 but were not determined in the NA subtype due to limitation of the extracted viral RNAs; the viruses diagnosed only as H5 subtype were expressed as H5Nx. Furthermore, among the 29 isolates from wild birds, 17, 10, and 2 viruses were confirmed from crows, raptors, and aquatic birds, respectively. All the AIV-positive crow and raptors were found within Hokkaido Island, where many of the migratory raptors are considered to travel. Two AIV-positive aquatic birds were found in the central-northern part of Japan (Fukushima and Iwate Prefs.), which are also expected to be destinations of bird migration from the continent Asia.

**Figure 2:**
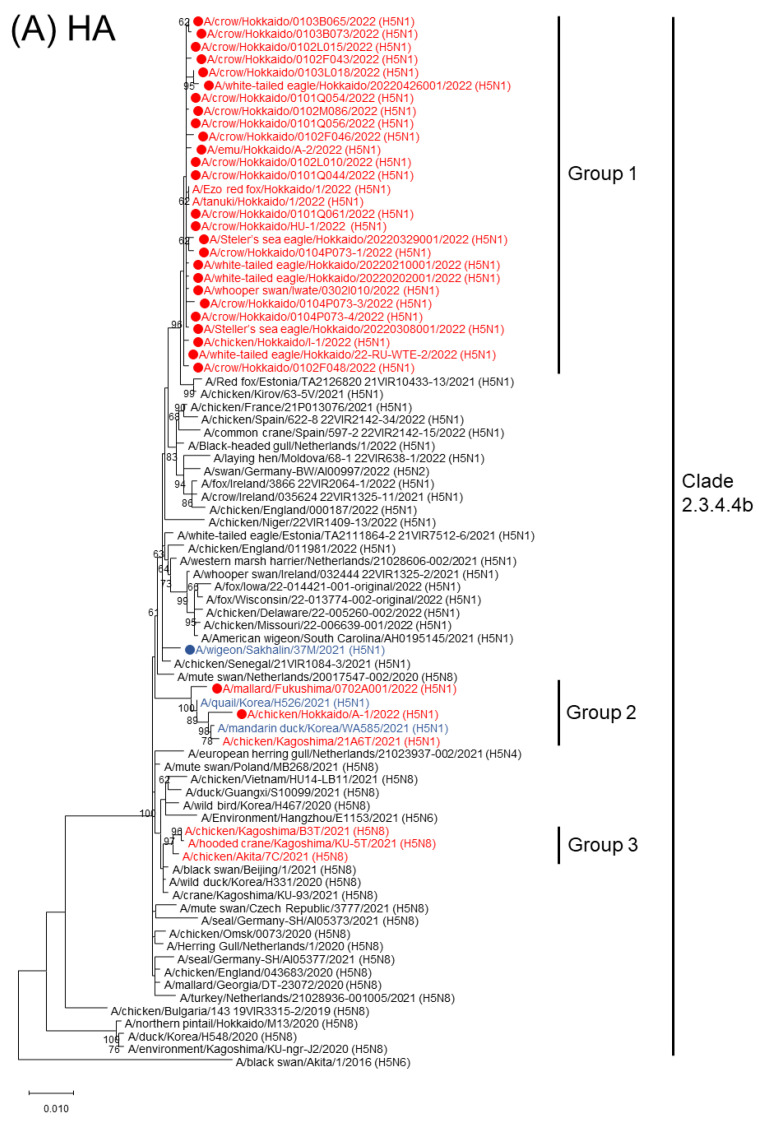

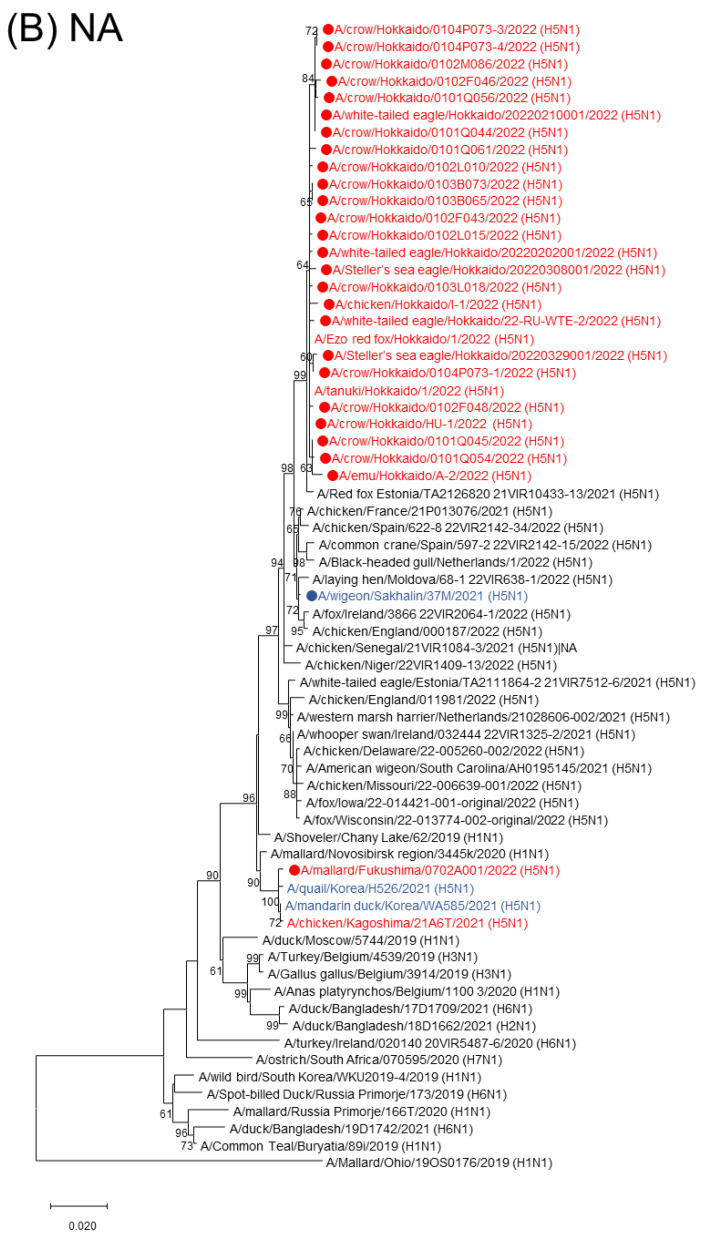
Phylogenetic tree analysis on the hemagglutinin (HA) and neuraminidase (NA) gene segment. Sequence data of the HA (**A**) and NA (**B**) genes of the viruses detected or isolated in this study and H5HA (**A**) and N1NA (**B**) viruses obtained from the Global Initiative on Sharing Avian Influenza Data database were used for constructing the phylogenetic tree. The red-colored strain indicates H5 HPAIVs detected or isolated in Japan in winter 2021–2022. The blue-colored strain indicates H5 HPAIVs isolated in the Republic of Korea or Sakhalin in the same season. The strain with a filled circle indicates the virus detected or isolated in this study. The numbers below or above the node indicate bootstrap values of >50%.

In the phylogenetic analysis using sequence data of 29 virus strains from wild birds in Japan, one strain isolated in a Russian wigeon and three strains from domestic poultry in Hokkaido, all 33 positives investigated were categorized into clade 2.3.4.4b sublineage of the H5 HA, and most of them formed the major genetic group (Group 1) while A/mallard/Fukushima/0702A001/2022 (H5N1) (Md/Fuk) and A/chicken/Hokkaido/A-1/2022 (H5N1) (Ck/Hok/A1), were classified into another genetic group (Group 2). H5 HPAIV isolates in the winter of 2021-2022 in Republic of Korea and the southern part of Japan were classified to two groups; Group 2 and Group 3. The viruses in Group 1 and 2 are genetically close to viruses prevalent in Europe in the winter 2021–2022 and summer in 2021, respectively, while viruses in Group 3 are genetically close to viruses in the Asia and in winter 2020–2021 (Figure 2). Nucleotide identity between the HA genes of representative strains of Group 1 and Group 2, Crow/Hok and Md/Fuk, respectively, was 98.32%. The phylogenetic tree of the N1 NA gene indicated that all H5N1 HPAIVs isolated in this study were almost identical and classified into the same cluster as those isolated in Europe in the same season as well. In phylogenetic trees of the other gene segments, a similar trend, as shown in the phylogenetic tree of the NA gene without any apparent distinctions on the classification of virus isolate, was confirmed (Supplemental Figure S1). BLAST analysis for two representative isolates, Crow/Hok and A/white-tailed eagle/Hokkaido/22-RU-WTE-2/2022 (H5N1) (WTE/Hok) indicated that all eight segments of both strains were most highly homologous to the corresponding gene segments of H5N1 HPAIVs isolated at the end of 2021 in Europe, including Sweden, Germany, and Romania (Table 1). These results could be correlated with phylogenetic trees of the other six gene segments as well as the HA and NA genes. In all phylogenetic trees of the six internal genes, all positive samples in this study formed one unique cluster next to European isolates in winter 2021–2022 (Supplemental Figure S1). Moreover, these viruses were also genetically similar to the one isolated in Sakhalin (Figure 2). In contrast, two positives, Md/Fuk and Ck/Hok/A1, were categorized into the cluster composed of viruses isolated at the end of 2021 in Japan and the Republic of Korea. These viruses are distinct from H5N8 HPAIV circulating in Asia in winter 2020–2021 but derived from those circulating in Europe in the same season.

**Figure 3:**
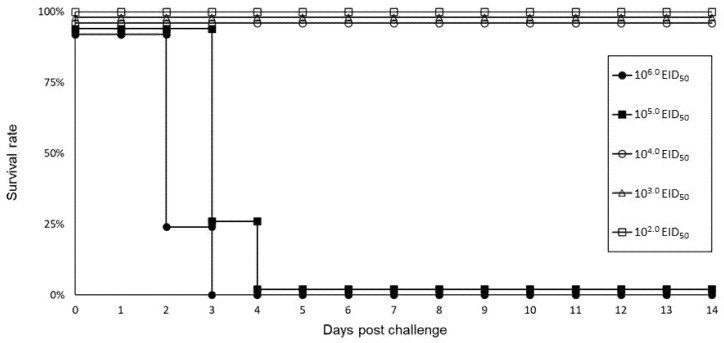
Survival curve of chickens intranasally inoculated with A/crow/Hokkaido/0103B065/2022 (H5N1). Five groups of four 6-week-old chickens were intranasally inoculated with 10^2.0^, 10^3.0^, 10^4.0^, 10^5.0^, and 10^6.0^ times of EID_50_ of A/crow/Hokkaido/0103B065/2022 (H5N1), respectively, and observed for 14 days after virus challenge. Clinical manifestations of chickens were observed, and their survivals were recorded daily to calculate the survival rate of each group per day.

To investigate the pathogenicity of a major strain of H5 HPAIVs isolated in Japan in winter 2021–2022, the representative strain, Crow/Hok, was intranasally inoculated into 6-week-old chickens with different virus titers. On 2 dpc, two chickens died, and one chicken showed apparent disease manifestations like depression and lack of appetite in the group of 10^6.0^ EID_50_ inoculation. On 3 dpc, all the remaining chickens in the group of 10^6.0^ EID_50_ inoculation died, and two and one chickens in the group of 10^5.0^ EID_50_ inoculation died and showed apparent disease manifestations. All chickens inoculated with 10^6.0^ or 10^5.0^ EID_50_ had died before 4 dpc, whereas birds inoculated with ≦10^4.0^ EID_50_ survived for 14 days after the challenge without any apparent clinical manifestations (Figure 3). The CLD_50_ of Crow/Hok in 6-week-old chickens was calculated as 10^4.5^ EID_50_.

## 4. Discussion

In winter 2021–2022, many H5N1 HPAIVs were introduced to the Far East, including Japan, Russia (Sakhalin), and the Republic of Korea [11,13]. Phylogenetic analysis results indicated that three groups of H5 HPAIVs were introduced into Japan, probably by bird migration. Most H5N1 isolates in northern Japan in Group 1 were genetically close in all eight gene segments to those circulating in Europe and North America in the same season, indicating that these viruses did not cause any genetic reassortment events with local viruses and had the potential to travel for a longer distance, such as from nested lakes in the north to the Far East along the Sea of Okhotsk as well as European countries [13], and were simultaneously introduced into the Far East, Europe, and North America [9,25]. In parallel, H5 HPAIVs isolated in southern Japan and the Republic of Korea at the end of 2021 were genetically distinct from Group 1 [13] and formed two different genetic subgroups in the phylogenetic tree of the HA gene (Group 2 and 3). Viruses in Group 3 were genetically close to Asian isolates in 2020–2021 [11,12,13], implying that H5 HPAIVs, mainly the H5H8 subtype in Group 3, had been kept in the Far East since the winter of 2020–2021. Viruses in Group 2 were genetically close to European isolates in summer or autumn 2021 but distinct from the viruses in Group 1. Given the four major routes of bird migration to Japan, it is reasonable to consider that HPAIVs had already been kept (Group 3) or transferred from Europe and been prevalent in summer 2021 (Group 2) and could be further brought to central or southern Japan and the Korean Peninsula. Thereafter, viruses in the Group 1 would be brought to northern Japan and circulated within Hokkaido Island by migratory birds. The difference in virus groups could be associated with the prevalent strains on the pathway of bird migration, indicating that various HPAIVs can potentially invade each part of Japan and could achieve domestic travel by bird migration. Invasion of another group of an HPAIV from majority in Hokkaido could be caused by bird migration to the north from the southern part of Japan, where HPAIVs genetically close to those circulating in the Republic of Korea or China were prevalent.

In the H5 HPAIV cases in winter 2021–2022, one of the unique features is that many dead crows were positive for H5 HPAIV, and approximately half of the positive viruses used in this study originated from crows. During the Japanese HPAIV outbreaks in 2004, many dead crows infected with HPAIV were reported [26], and the high sensitivity of crows to HPAIVs was confirmed in the experimental setting [27]. In addition, crows are considered to play a role in virus transmission from outside to inside of the poultry farm. Nevertheless, the unusual number of HPAI cases in crows, and probably raptors as well, might imply the potential change of the HPAIV circulation mechanism and its ecology in the field. To assume the introduction of contagious viruses from outside to inside of poultry farms by wild birds, an evaluation of the pathogenicity of an HPAIV isolated from the crow in chickens was necessary to investigate how this potential change in viral ecology could influence HPAI epidemiology in poultry. In this experimental infection, all chickens intranasally challenged with 10^6.0^ and 10^5.0^ EID_50_ of Crow/Hok died until 3 and 4 dpi, respectively. The CLD_50_ of Crow/Hok was 10^4.5^ EID_50_ and comparative to H5 HPAIV isolates between 2014 and 2020 [6], indicating that pathogenicity of the Crow/Hok would not be so different from the contagious HPAIV invading Japan in the last decade although no statistical analyses were conducted due to a lack of survival data in past reports. Like crows, many debilitated or dead raptors were found in winter 2021–2022 and diagnosed as positive for H5N1 HPAIV infections. Past reports of HPAIV infections in eagles could support the proposition that eagles and other raptors have the potential to be infected with HPAIVs, probably via preying on diseased birds [28,29].

One of the outstanding considerations during the HPAIV cases in winter 2021–2022 was the smaller proportion of HPAIV infection cases in waterfowl, which would usually be the preys of raptors, compared to the last few years [17]. Both of the two H5 HPAIV positive cases in waterfowl were not confirmed by virus isolation but by direct sequencing. One of the strong points in the updated definite diagnosis protocol of HPAIV infections in wild birds should be the capability to diagnose infected birds with HPAIV but mild pathogenicity, which would shed limited numbers of infectious viruses. Moreover, detecting a low amount of HPAIV genes should be important to pick up minor infections to grab a more accurate overview of HPAIV circulation and understand the entire HPAI ecology. Case reports of HPAIV infection in waterfowl were also limited in Europe, probably due to the lower pathogenicity of currently circulating HPAIVs to duck species, as revealed by experimental infections in chickens and Peking ducks [30]. Because waterfowls, like duck species, are the main drivers of HPAIV transmission, the ecological and epidemiological change of HPAIV in waterfowl would seriously affect the entire disease dynamics. Lower pathogenicity in ducks could explain the possibility of longer travel of HPAIVs by bird migration; thus, continuous disease spread at the global level would be ensured. Furthermore, it may warn the perpetuation of HPAIVs in nested lakes of migratory waterfowl by delivering contagious viruses via bird migration. Active surveillance for migratory birds from the north should be essential to assess the proposition and estimate the risk of intercontinental movement of HPAIVs.

Given the tremendous numbers of HPAIV infections potentially spread by bird migration in multiple regions, poultry farms should strengthen biosecurity measures to prevent the risk of HPAI outbreaks in poultry from wild birds and save the poultry industry. Indeed, high homology in the genetic sequences between HPAIVs from wild birds in local and poultry in Hokkaido could support the horizontal transmission between them, whereas one accidental virus introduction to the poultry farm from a far-distanced area was suspected. In addition, the international community should work to reduce the risk of HPAIV dissemination through bird migration, such as establishing a global virus monitoring system.

## Figures and Tables

**Table 1:** BLAST search results of nucleotide sequence of two representative strains in the GISAID database.

Virus	Gene	Most Homologous Strain	Homology	Accession Number
A/white-tailed eagle/Hokkaido/22-RU-WTE-2/2022 (H5N1)	PB2	A/buzzard/Germany-SH/AI07099/2021 (H5N1)	99.74%	EPI2009974
	PB1	A/buzzard/Germany-SH/AI07099/2021 (H5N1)	99.74%	EPI2009973
	PA	A/white-tailed eagle/Sweden/SVA211201SZ0380/FB004721/M-2021 (H5N1)	99.59%	EPI1943097
	HA	A/buzzard/Germany-SH/AI07099/2021 (H5N1)	99.60%	EPI2009967
	NP	A/buzzard/Germany-SH/AI07099/2021 (H5N1)	99.74%	EPI2009970
	NA	A/greylag goose /Sweden/SVA211111SZ0376/FB004497/M-2021 (H5N1)	99.65%	EPI1938854
	M	A/swan/Romania/10986 22VIR2749-8/2022 (H5N1)	99.60%	EPI2015036
	NS	A/greylag goose/Denmark/24309-1.01/2021-10-27 (H5N1)	99.77%	EPI2015081
A/crow/Hokkaido/0103B065/2022 (H5N1)	PB2	A/greylag goose /Sweden/SVA211111SZ0376/FB004497/M-2021 (H5N1)	99.74%	EPI1938849
	PB1	A/white-tailed eagle/Sweden/SVA211201SZ0380/FB004721/M-2021 (H5N1)	99.66%	EPI1943096
	PA	A/white-tailed eagle/Sweden/SVA211201SZ0380/FB004721/M-2021 (H5N1)	99.64%	EPI1943097
	HA	A/chicken/Kirov/63-5V/2021 (H5N1)	99.49%	EPI1958041
	NP	A/white-tailed eagle/Sweden/SVA211201SZ0380/FB004721/M-2021 (H5N1)	99.74%	EPI1943099
	NA	A/greylag goose /Sweden/SVA211111SZ0376/FB004497/M-2021 (H5N1)	99.72%	EPI1938854
	M	A/swan/Romania/10986 22VIR2749-8/2022 (H5N1)	99.80%	EPI2015036
	NS	A/barnacle goose/Sweden/SVA211111SZ0376/FB004496/2021 (H5N1)	100.00%	EPI1938848

## Data Availability

The data presented in this study are openly available in the Global Initiative on Sharing All Influenza Data and the DNA data bank of Japan. All the accession numbers of the virus sequence data were described in Table S1.

## Supplementary Materials

The following supporting information can be downloaded at: https://www.mdpi.com/article/10.3390/v14102168/s1, Figure S1: Phylogenetic tree analysis on the internal gene segments (PB2, PB1, PA, NP, M, NS), Table S1: List of H5 high pathogenicity avian influenza viruses isolated or detected in the current study.
