# Supplementary material for: Detection of New H5N1 High Pathogenicity Avian Influenza Viruses in Winter 2021–2022 in the Far East, Which Are Genetically Close to Those in Europe
Source: Viruses. 2022 Sep 30;14(10):2168. doi: 10.3390/v14102168 (PMC9606862; doi:10.3390/v14102168)
Supplement: Supplementary file 1 [file viruses-14-02168-s001.zip › viruses-1916543-supplementary.pptx]

## Slide 1
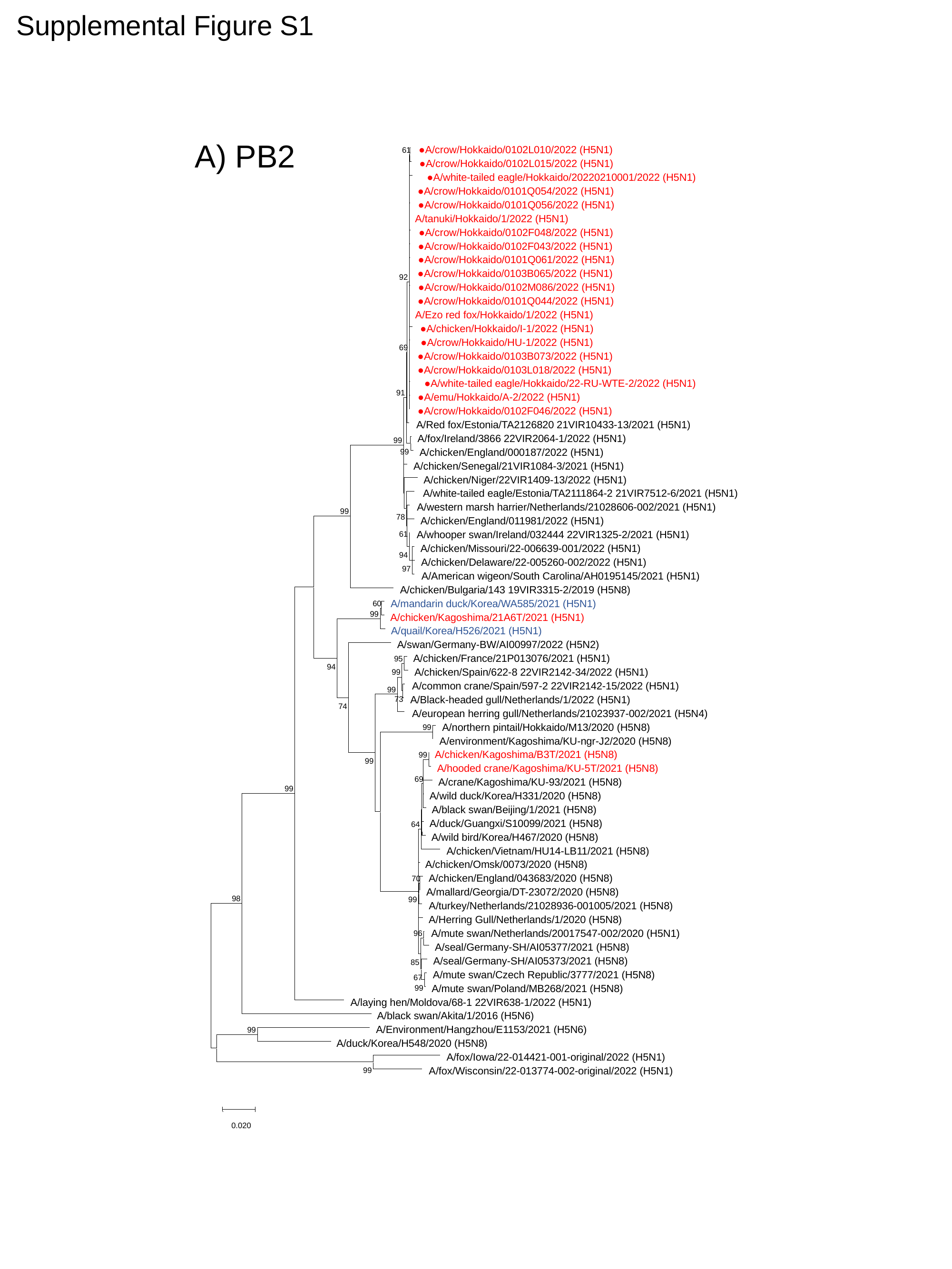

Supplemental Figure S1
 ●A/crow/Hokkaido/0102L010/2022 (H5N1)
 ●A/crow/Hokkaido/0102L015/2022 (H5N1)
 ●A/white-tailed eagle/Hokkaido/20220210001/2022 (H5N1)
 ●A/crow/Hokkaido/0101Q054/2022 (H5N1)
 ●A/crow/Hokkaido/0101Q056/2022 (H5N1)
 A/tanuki/Hokkaido/1/2022 (H5N1)
 ●A/crow/Hokkaido/0102F048/2022 (H5N1)
 ●A/crow/Hokkaido/0102F043/2022 (H5N1)
 ●A/crow/Hokkaido/0101Q061/2022 (H5N1)
 ●A/crow/Hokkaido/0103B065/2022 (H5N1)
 ●A/crow/Hokkaido/0102M086/2022 (H5N1)
 ●A/crow/Hokkaido/0101Q044/2022 (H5N1)
 A/Ezo red fox/Hokkaido/1/2022 (H5N1)
 ●A/chicken/Hokkaido/I-1/2022 (H5N1)
 ●A/crow/Hokkaido/HU-1/2022 (H5N1)
 ●A/crow/Hokkaido/0103B073/2022 (H5N1)
 ●A/crow/Hokkaido/0103L018/2022 (H5N1)
 ●A/white-tailed eagle/Hokkaido/22-RU-WTE-2/2022 (H5N1)
 ●A/emu/Hokkaido/A-2/2022 (H5N1)
 ●A/crow/Hokkaido/0102F046/2022 (H5N1)
 A/Red fox/Estonia/TA2126820 21VIR10433-13/2021 (H5N1)
 A/fox/Ireland/3866 22VIR2064-1/2022 (H5N1)
 A/chicken/England/000187/2022 (H5N1)
 A/chicken/Senegal/21VIR1084-3/2021 (H5N1)
 A/chicken/Niger/22VIR1409-13/2022 (H5N1)
 A/white-tailed eagle/Estonia/TA2111864-2 21VIR7512-6/2021 (H5N1)
 A/western marsh harrier/Netherlands/21028606-002/2021 (H5N1)
 A/chicken/England/011981/2022 (H5N1)
 A/whooper swan/Ireland/032444 22VIR1325-2/2021 (H5N1)
 A/chicken/Missouri/22-006639-001/2022 (H5N1)
 A/chicken/Delaware/22-005260-002/2022 (H5N1)
 A/American wigeon/South Carolina/AH0195145/2021 (H5N1)
 A/chicken/Bulgaria/143 19VIR3315-2/2019 (H5N8)
 A/mandarin duck/Korea/WA585/2021 (H5N1)
60
99
 A/chicken/Kagoshima/21A6T/2021 (H5N1)
 A/quail/Korea/H526/2021 (H5N1)
 A/swan/Germany-BW/AI00997/2022 (H5N2)
 A/chicken/France/21P013076/2021 (H5N1)
 A/chicken/Spain/622-8 22VIR2142-34/2022 (H5N1)
 A/common crane/Spain/597-2 22VIR2142-15/2022 (H5N1)
 A/Black-headed gull/Netherlands/1/2022 (H5N1)
 A/european herring gull/Netherlands/21023937-002/2021 (H5N4)
 A/northern pintail/Hokkaido/M13/2020 (H5N8)
99
 A/environment/Kagoshima/KU-ngr-J2/2020 (H5N8)
 A/chicken/Kagoshima/B3T/2021 (H5N8)
99
 A/hooded crane/Kagoshima/KU-5T/2021 (H5N8)
69
 A/crane/Kagoshima/KU-93/2021 (H5N8)
 A/wild duck/Korea/H331/2020 (H5N8)
 A/black swan/Beijing/1/2021 (H5N8)
 A/duck/Guangxi/S10099/2021 (H5N8)
 A/wild bird/Korea/H467/2020 (H5N8)
 A/chicken/Vietnam/HU14-LB11/2021 (H5N8)
 A/chicken/Omsk/0073/2020 (H5N8)
 A/chicken/England/043683/2020 (H5N8)
70
 A/mallard/Georgia/DT-23072/2020 (H5N8)
99
 A/turkey/Netherlands/21028936-001005/2021 (H5N8)
 A/Herring Gull/Netherlands/1/2020 (H5N8)
 A/mute swan/Netherlands/20017547-002/2020 (H5N1)
96
 A/seal/Germany-SH/AI05377/2021 (H5N8)
 A/seal/Germany-SH/AI05373/2021 (H5N8)
85
 A/mute swan/Czech Republic/3777/2021 (H5N8)
67
 A/mute swan/Poland/MB268/2021 (H5N8)
99
 A/laying hen/Moldova/68-1 22VIR638-1/2022 (H5N1)
 A/black swan/Akita/1/2016 (H5N6)
 A/Environment/Hangzhou/E1153/2021 (H5N6)
99
 A/duck/Korea/H548/2020 (H5N8)
 A/fox/Iowa/22-014421-001-original/2022 (H5N1)
 A/fox/Wisconsin/22-013774-002-original/2022 (H5N1)
99
61
92
69
91
99
99
99
78
61
94
97
95
94
99
99
73
74
99
99
64
98
0.020
A) PB2

## Slide 2
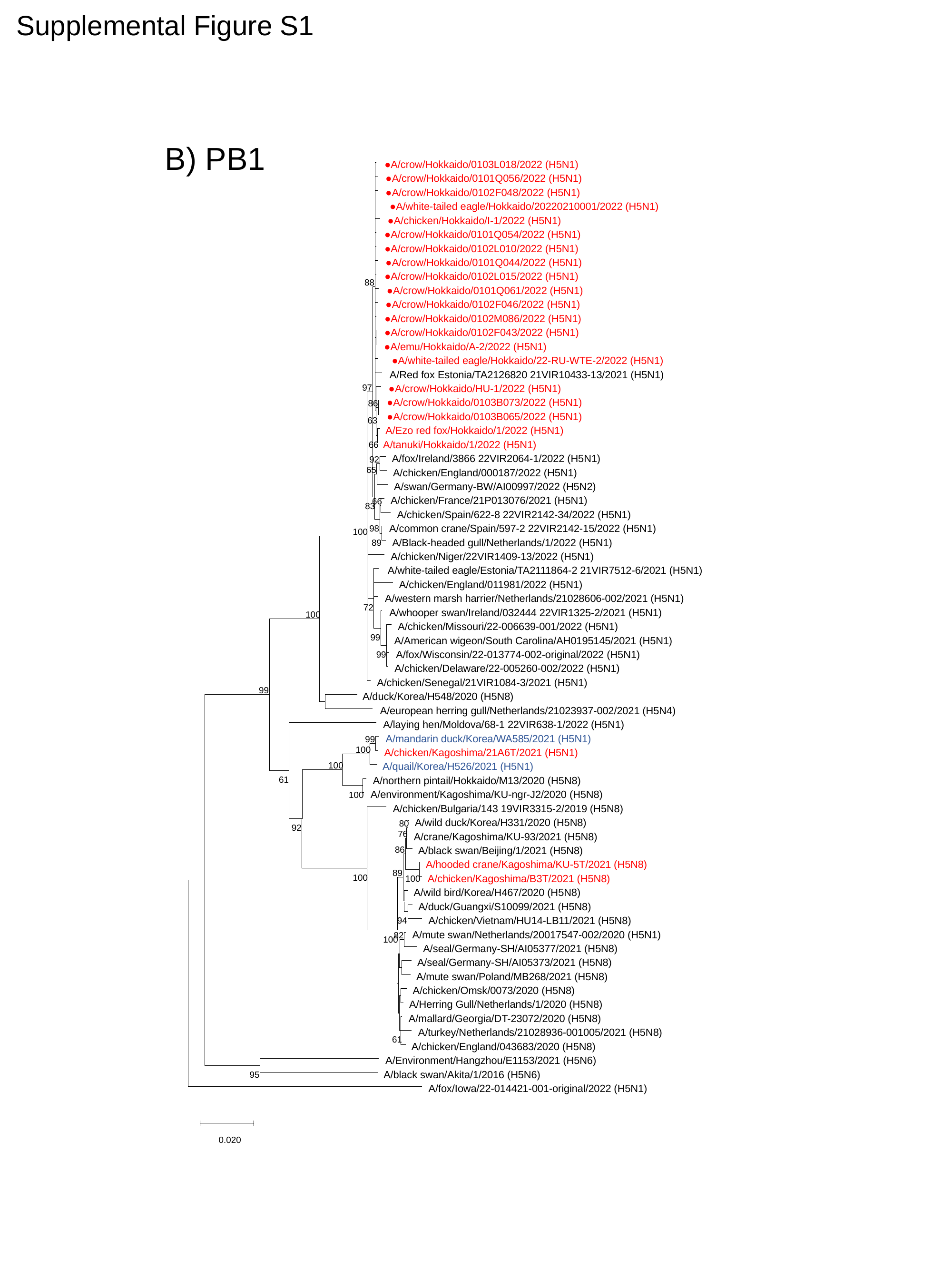

Supplemental Figure S1
B) PB1
 ●A/crow/Hokkaido/0103L018/2022 (H5N1)
 ●A/crow/Hokkaido/0101Q056/2022 (H5N1)
 ●A/crow/Hokkaido/0102F048/2022 (H5N1)
 ●A/white-tailed eagle/Hokkaido/20220210001/2022 (H5N1)
 ●A/chicken/Hokkaido/I-1/2022 (H5N1)
 ●A/crow/Hokkaido/0101Q054/2022 (H5N1)
 ●A/crow/Hokkaido/0102L010/2022 (H5N1)
 ●A/crow/Hokkaido/0101Q044/2022 (H5N1)
 ●A/crow/Hokkaido/0102L015/2022 (H5N1)
 ●A/crow/Hokkaido/0101Q061/2022 (H5N1)
 ●A/crow/Hokkaido/0102F046/2022 (H5N1)
 ●A/crow/Hokkaido/0102M086/2022 (H5N1)
 ●A/crow/Hokkaido/0102F043/2022 (H5N1)
 ●A/emu/Hokkaido/A-2/2022 (H5N1)
 ●A/white-tailed eagle/Hokkaido/22-RU-WTE-2/2022 (H5N1)
 A/Red fox Estonia/TA2126820 21VIR10433-13/2021 (H5N1)
 ●A/crow/Hokkaido/HU-1/2022 (H5N1)
 ●A/crow/Hokkaido/0103B073/2022 (H5N1)
 ●A/crow/Hokkaido/0103B065/2022 (H5N1)
 A/Ezo red fox/Hokkaido/1/2022 (H5N1)
 A/tanuki/Hokkaido/1/2022 (H5N1)
 A/fox/Ireland/3866 22VIR2064-1/2022 (H5N1)
 A/chicken/England/000187/2022 (H5N1)
 A/swan/Germany-BW/AI00997/2022 (H5N2)
 A/chicken/France/21P013076/2021 (H5N1)
 A/chicken/Spain/622-8 22VIR2142-34/2022 (H5N1)
 A/common crane/Spain/597-2 22VIR2142-15/2022 (H5N1)
 A/Black-headed gull/Netherlands/1/2022 (H5N1)
 A/chicken/Niger/22VIR1409-13/2022 (H5N1)
 A/white-tailed eagle/Estonia/TA2111864-2 21VIR7512-6/2021 (H5N1)
 A/chicken/England/011981/2022 (H5N1)
 A/western marsh harrier/Netherlands/21028606-002/2021 (H5N1)
 A/whooper swan/Ireland/032444 22VIR1325-2/2021 (H5N1)
 A/chicken/Missouri/22-006639-001/2022 (H5N1)
 A/American wigeon/South Carolina/AH0195145/2021 (H5N1)
 A/fox/Wisconsin/22-013774-002-original/2022 (H5N1)
 A/chicken/Delaware/22-005260-002/2022 (H5N1)
 A/chicken/Senegal/21VIR1084-3/2021 (H5N1)
 A/duck/Korea/H548/2020 (H5N8)
 A/european herring gull/Netherlands/21023937-002/2021 (H5N4)
 A/laying hen/Moldova/68-1 22VIR638-1/2022 (H5N1)
 A/mandarin duck/Korea/WA585/2021 (H5N1)
99
100
 A/chicken/Kagoshima/21A6T/2021 (H5N1)
 A/quail/Korea/H526/2021 (H5N1)
 A/northern pintail/Hokkaido/M13/2020 (H5N8)
 A/environment/Kagoshima/KU-ngr-J2/2020 (H5N8)
100
 A/chicken/Bulgaria/143 19VIR3315-2/2019 (H5N8)
 A/wild duck/Korea/H331/2020 (H5N8)
80
76
 A/crane/Kagoshima/KU-93/2021 (H5N8)
86
 A/black swan/Beijing/1/2021 (H5N8)
 A/hooded crane/Kagoshima/KU-5T/2021 (H5N8)
89
 A/chicken/Kagoshima/B3T/2021 (H5N8)
100
 A/wild bird/Korea/H467/2020 (H5N8)
 A/duck/Guangxi/S10099/2021 (H5N8)
 A/chicken/Vietnam/HU14-LB11/2021 (H5N8)
94
 A/mute swan/Netherlands/20017547-002/2020 (H5N1)
82
 A/seal/Germany-SH/AI05377/2021 (H5N8)
 A/seal/Germany-SH/AI05373/2021 (H5N8)
 A/mute swan/Poland/MB268/2021 (H5N8)
 A/chicken/Omsk/0073/2020 (H5N8)
 A/Herring Gull/Netherlands/1/2020 (H5N8)
 A/mallard/Georgia/DT-23072/2020 (H5N8)
 A/turkey/Netherlands/21028936-001005/2021 (H5N8)
61
 A/chicken/England/043683/2020 (H5N8)
 A/Environment/Hangzhou/E1153/2021 (H5N6)
 A/black swan/Akita/1/2016 (H5N6)
95
 A/fox/Iowa/22-014421-001-original/2022 (H5N1)
88
97
86
63
66
92
65
66
83
98
100
89
72
100
99
99
99
100
61
92
100
100
0.020

## Slide 3
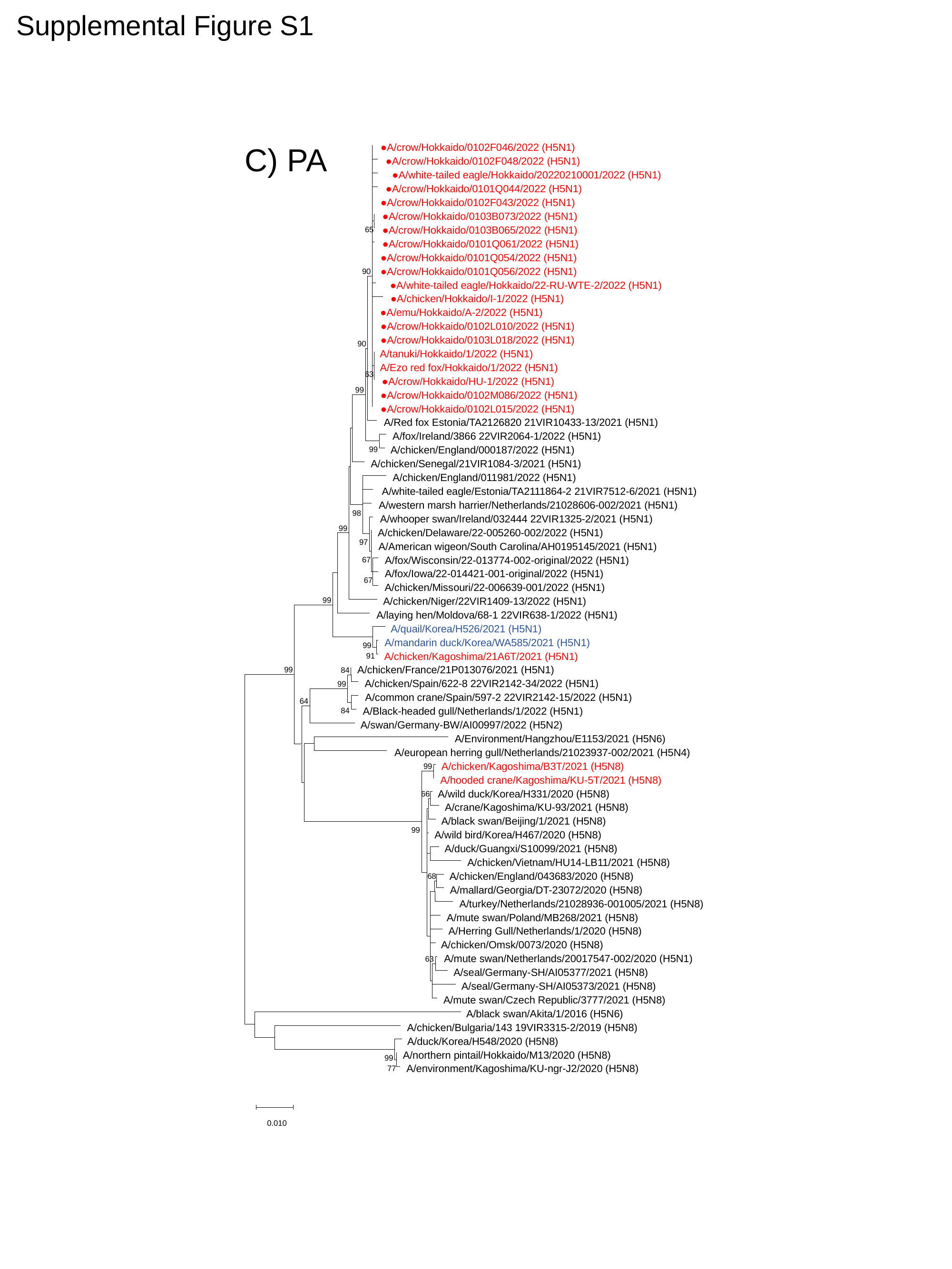

Supplemental Figure S1
C) PA
 ●A/crow/Hokkaido/0102F046/2022 (H5N1)
 ●A/crow/Hokkaido/0102F048/2022 (H5N1)
 ●A/white-tailed eagle/Hokkaido/20220210001/2022 (H5N1)
 ●A/crow/Hokkaido/0101Q044/2022 (H5N1)
 ●A/crow/Hokkaido/0102F043/2022 (H5N1)
 ●A/crow/Hokkaido/0103B073/2022 (H5N1)
 ●A/crow/Hokkaido/0103B065/2022 (H5N1)
 ●A/crow/Hokkaido/0101Q061/2022 (H5N1)
 ●A/crow/Hokkaido/0101Q054/2022 (H5N1)
 ●A/crow/Hokkaido/0101Q056/2022 (H5N1)
 ●A/white-tailed eagle/Hokkaido/22-RU-WTE-2/2022 (H5N1)
 ●A/chicken/Hokkaido/I-1/2022 (H5N1)
 ●A/emu/Hokkaido/A-2/2022 (H5N1)
 ●A/crow/Hokkaido/0102L010/2022 (H5N1)
 ●A/crow/Hokkaido/0103L018/2022 (H5N1)
 A/tanuki/Hokkaido/1/2022 (H5N1)
 A/Ezo red fox/Hokkaido/1/2022 (H5N1)
 ●A/crow/Hokkaido/HU-1/2022 (H5N1)
 ●A/crow/Hokkaido/0102M086/2022 (H5N1)
 ●A/crow/Hokkaido/0102L015/2022 (H5N1)
 A/Red fox Estonia/TA2126820 21VIR10433-13/2021 (H5N1)
 A/fox/Ireland/3866 22VIR2064-1/2022 (H5N1)
 A/chicken/England/000187/2022 (H5N1)
 A/chicken/Senegal/21VIR1084-3/2021 (H5N1)
 A/chicken/England/011981/2022 (H5N1)
 A/white-tailed eagle/Estonia/TA2111864-2 21VIR7512-6/2021 (H5N1)
 A/western marsh harrier/Netherlands/21028606-002/2021 (H5N1)
 A/whooper swan/Ireland/032444 22VIR1325-2/2021 (H5N1)
 A/chicken/Delaware/22-005260-002/2022 (H5N1)
 A/American wigeon/South Carolina/AH0195145/2021 (H5N1)
 A/fox/Wisconsin/22-013774-002-original/2022 (H5N1)
 A/fox/Iowa/22-014421-001-original/2022 (H5N1)
 A/chicken/Missouri/22-006639-001/2022 (H5N1)
 A/chicken/Niger/22VIR1409-13/2022 (H5N1)
 A/laying hen/Moldova/68-1 22VIR638-1/2022 (H5N1)
 A/quail/Korea/H526/2021 (H5N1)
 A/mandarin duck/Korea/WA585/2021 (H5N1)
 A/chicken/Kagoshima/21A6T/2021 (H5N1)
 A/chicken/France/21P013076/2021 (H5N1)
 A/chicken/Spain/622-8 22VIR2142-34/2022 (H5N1)
 A/common crane/Spain/597-2 22VIR2142-15/2022 (H5N1)
 A/Black-headed gull/Netherlands/1/2022 (H5N1)
 A/swan/Germany-BW/AI00997/2022 (H5N2)
 A/Environment/Hangzhou/E1153/2021 (H5N6)
 A/european herring gull/Netherlands/21023937-002/2021 (H5N4)
 A/chicken/Kagoshima/B3T/2021 (H5N8)
99
 A/hooded crane/Kagoshima/KU-5T/2021 (H5N8)
 A/wild duck/Korea/H331/2020 (H5N8)
66
 A/crane/Kagoshima/KU-93/2021 (H5N8)
 A/black swan/Beijing/1/2021 (H5N8)
99
 A/wild bird/Korea/H467/2020 (H5N8)
 A/duck/Guangxi/S10099/2021 (H5N8)
 A/chicken/Vietnam/HU14-LB11/2021 (H5N8)
 A/chicken/England/043683/2020 (H5N8)
68
 A/mallard/Georgia/DT-23072/2020 (H5N8)
 A/turkey/Netherlands/21028936-001005/2021 (H5N8)
 A/mute swan/Poland/MB268/2021 (H5N8)
 A/Herring Gull/Netherlands/1/2020 (H5N8)
 A/chicken/Omsk/0073/2020 (H5N8)
 A/mute swan/Netherlands/20017547-002/2020 (H5N1)
63
 A/seal/Germany-SH/AI05377/2021 (H5N8)
 A/seal/Germany-SH/AI05373/2021 (H5N8)
 A/mute swan/Czech Republic/3777/2021 (H5N8)
 A/black swan/Akita/1/2016 (H5N6)
 A/chicken/Bulgaria/143 19VIR3315-2/2019 (H5N8)
 A/duck/Korea/H548/2020 (H5N8)
 A/northern pintail/Hokkaido/M13/2020 (H5N8)
99
 A/environment/Kagoshima/KU-ngr-J2/2020 (H5N8)
77
65
90
90
63
99
99
98
99
97
67
67
99
99
91
99
84
99
64
84
0.010

## Slide 4
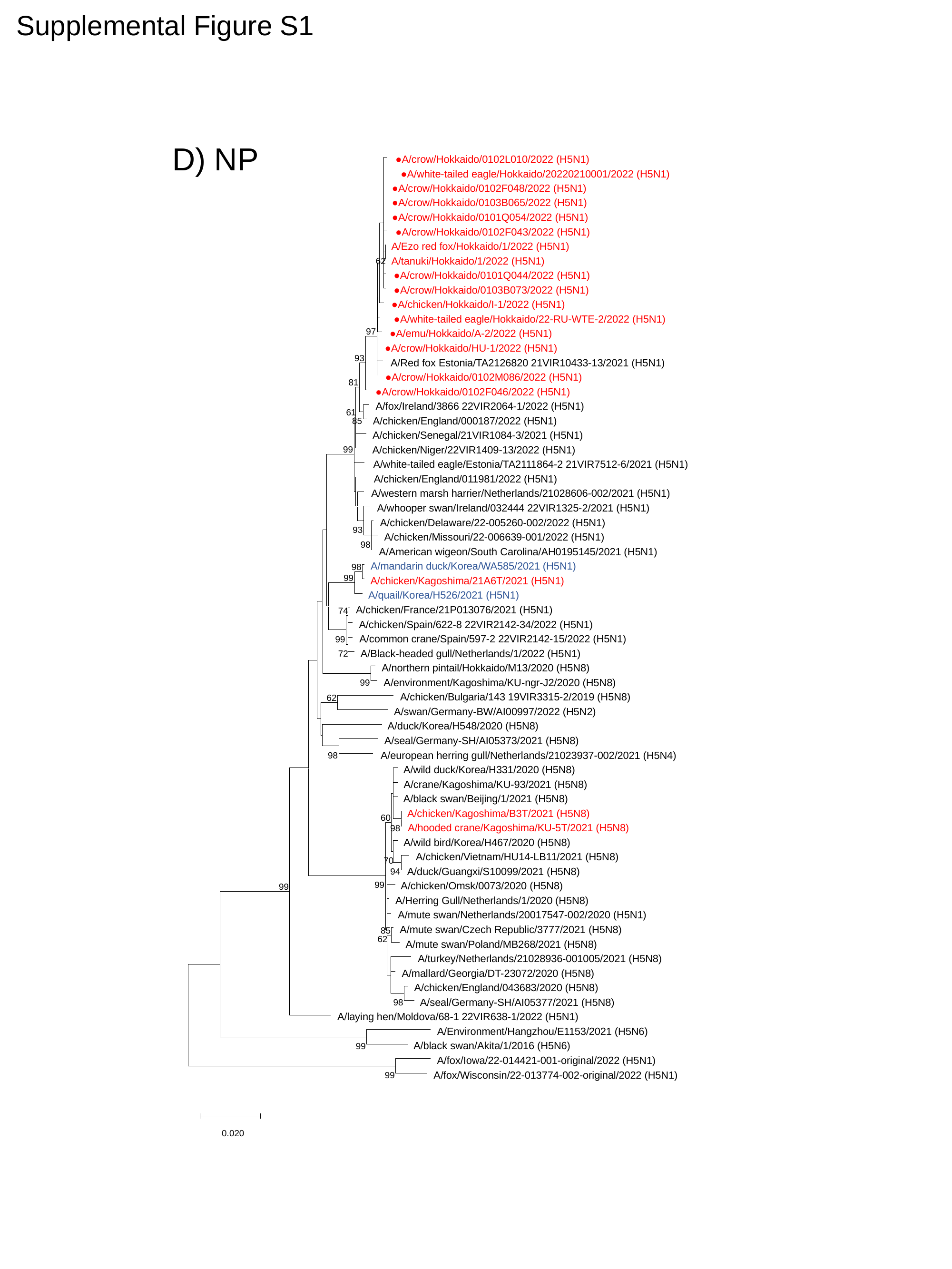

Supplemental Figure S1
D) NP
 ●A/crow/Hokkaido/0102L010/2022 (H5N1)
 ●A/white-tailed eagle/Hokkaido/20220210001/2022 (H5N1)
 ●A/crow/Hokkaido/0102F048/2022 (H5N1)
 ●A/crow/Hokkaido/0103B065/2022 (H5N1)
 ●A/crow/Hokkaido/0101Q054/2022 (H5N1)
 ●A/crow/Hokkaido/0102F043/2022 (H5N1)
 A/Ezo red fox/Hokkaido/1/2022 (H5N1)
 A/tanuki/Hokkaido/1/2022 (H5N1)
 ●A/crow/Hokkaido/0101Q044/2022 (H5N1)
 ●A/crow/Hokkaido/0103B073/2022 (H5N1)
 ●A/chicken/Hokkaido/I-1/2022 (H5N1)
 ●A/white-tailed eagle/Hokkaido/22-RU-WTE-2/2022 (H5N1)
 ●A/emu/Hokkaido/A-2/2022 (H5N1)
 ●A/crow/Hokkaido/HU-1/2022 (H5N1)
 A/Red fox Estonia/TA2126820 21VIR10433-13/2021 (H5N1)
 ●A/crow/Hokkaido/0102M086/2022 (H5N1)
 ●A/crow/Hokkaido/0102F046/2022 (H5N1)
 A/fox/Ireland/3866 22VIR2064-1/2022 (H5N1)
 A/chicken/England/000187/2022 (H5N1)
 A/chicken/Senegal/21VIR1084-3/2021 (H5N1)
 A/chicken/Niger/22VIR1409-13/2022 (H5N1)
 A/white-tailed eagle/Estonia/TA2111864-2 21VIR7512-6/2021 (H5N1)
 A/chicken/England/011981/2022 (H5N1)
 A/western marsh harrier/Netherlands/21028606-002/2021 (H5N1)
 A/whooper swan/Ireland/032444 22VIR1325-2/2021 (H5N1)
 A/chicken/Delaware/22-005260-002/2022 (H5N1)
 A/chicken/Missouri/22-006639-001/2022 (H5N1)
 A/American wigeon/South Carolina/AH0195145/2021 (H5N1)
 A/mandarin duck/Korea/WA585/2021 (H5N1)
98
99
 A/chicken/Kagoshima/21A6T/2021 (H5N1)
 A/quail/Korea/H526/2021 (H5N1)
 A/chicken/France/21P013076/2021 (H5N1)
 A/chicken/Spain/622-8 22VIR2142-34/2022 (H5N1)
 A/common crane/Spain/597-2 22VIR2142-15/2022 (H5N1)
 A/Black-headed gull/Netherlands/1/2022 (H5N1)
 A/northern pintail/Hokkaido/M13/2020 (H5N8)
 A/environment/Kagoshima/KU-ngr-J2/2020 (H5N8)
99
 A/chicken/Bulgaria/143 19VIR3315-2/2019 (H5N8)
62
 A/swan/Germany-BW/AI00997/2022 (H5N2)
 A/duck/Korea/H548/2020 (H5N8)
 A/seal/Germany-SH/AI05373/2021 (H5N8)
 A/european herring gull/Netherlands/21023937-002/2021 (H5N4)
98
 A/wild duck/Korea/H331/2020 (H5N8)
 A/crane/Kagoshima/KU-93/2021 (H5N8)
 A/black swan/Beijing/1/2021 (H5N8)
 A/chicken/Kagoshima/B3T/2021 (H5N8)
60
 A/hooded crane/Kagoshima/KU-5T/2021 (H5N8)
98
 A/wild bird/Korea/H467/2020 (H5N8)
 A/chicken/Vietnam/HU14-LB11/2021 (H5N8)
70
 A/duck/Guangxi/S10099/2021 (H5N8)
94
99
 A/chicken/Omsk/0073/2020 (H5N8)
 A/Herring Gull/Netherlands/1/2020 (H5N8)
 A/mute swan/Netherlands/20017547-002/2020 (H5N1)
 A/mute swan/Czech Republic/3777/2021 (H5N8)
85
62
 A/mute swan/Poland/MB268/2021 (H5N8)
 A/turkey/Netherlands/21028936-001005/2021 (H5N8)
 A/mallard/Georgia/DT-23072/2020 (H5N8)
 A/chicken/England/043683/2020 (H5N8)
 A/seal/Germany-SH/AI05377/2021 (H5N8)
98
 A/laying hen/Moldova/68-1 22VIR638-1/2022 (H5N1)
 A/Environment/Hangzhou/E1153/2021 (H5N6)
 A/black swan/Akita/1/2016 (H5N6)
99
 A/fox/Iowa/22-014421-001-original/2022 (H5N1)
 A/fox/Wisconsin/22-013774-002-original/2022 (H5N1)
99
62
97
93
81
61
85
99
93
98
74
99
72
99
0.020

## Slide 5
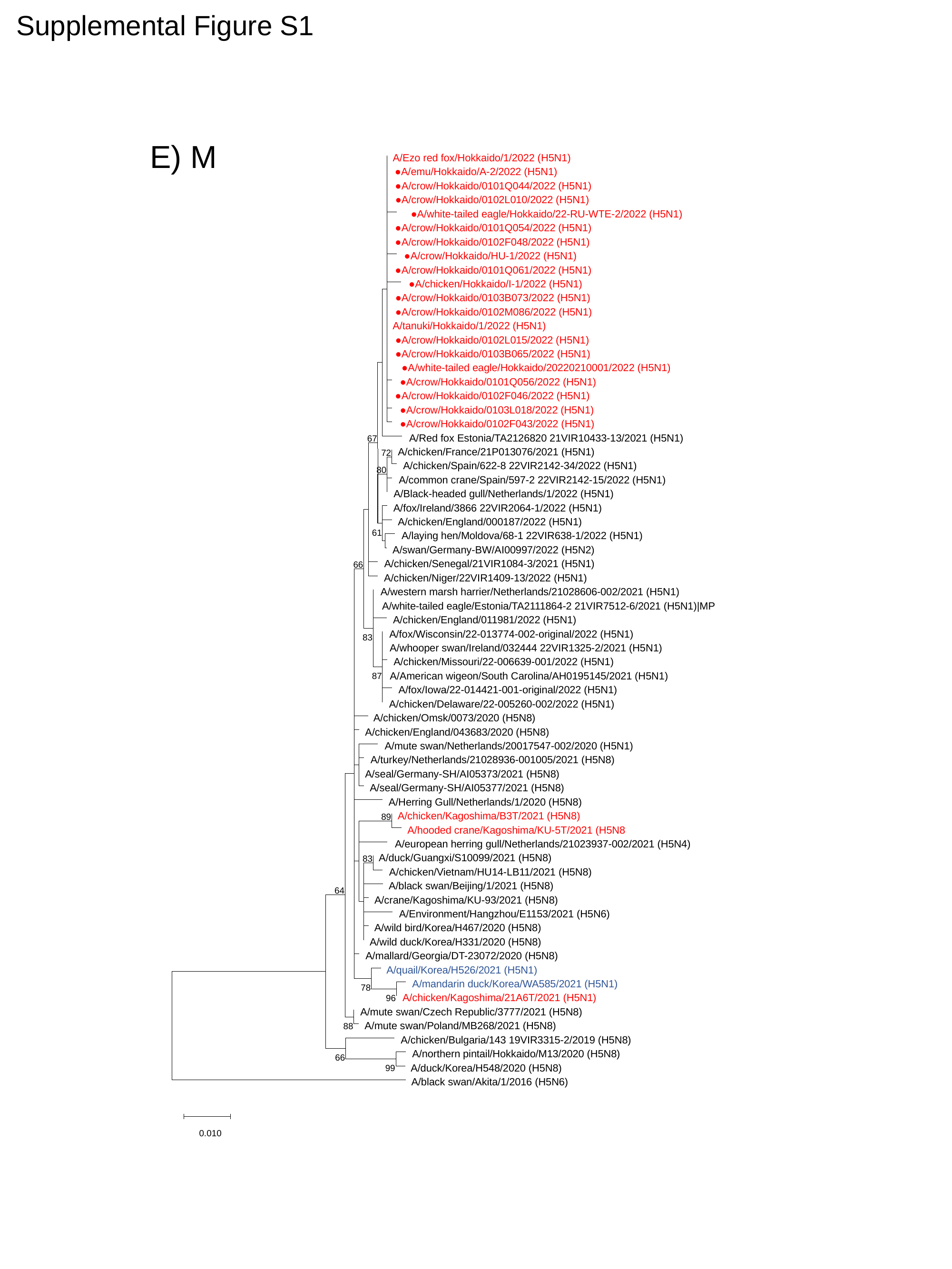

Supplemental Figure S1
E) M
 A/Ezo red fox/Hokkaido/1/2022 (H5N1)
 ●A/emu/Hokkaido/A-2/2022 (H5N1)
 ●A/crow/Hokkaido/0101Q044/2022 (H5N1)
 ●A/crow/Hokkaido/0102L010/2022 (H5N1)
 ●A/white-tailed eagle/Hokkaido/22-RU-WTE-2/2022 (H5N1)
 ●A/crow/Hokkaido/0101Q054/2022 (H5N1)
 ●A/crow/Hokkaido/0102F048/2022 (H5N1)
 ●A/crow/Hokkaido/HU-1/2022 (H5N1)
 ●A/crow/Hokkaido/0101Q061/2022 (H5N1)
 ●A/chicken/Hokkaido/I-1/2022 (H5N1)
 ●A/crow/Hokkaido/0103B073/2022 (H5N1)
 ●A/crow/Hokkaido/0102M086/2022 (H5N1)
 A/tanuki/Hokkaido/1/2022 (H5N1)
 ●A/crow/Hokkaido/0102L015/2022 (H5N1)
 ●A/crow/Hokkaido/0103B065/2022 (H5N1)
 ●A/white-tailed eagle/Hokkaido/20220210001/2022 (H5N1)
 ●A/crow/Hokkaido/0101Q056/2022 (H5N1)
 ●A/crow/Hokkaido/0102F046/2022 (H5N1)
 ●A/crow/Hokkaido/0103L018/2022 (H5N1)
 ●A/crow/Hokkaido/0102F043/2022 (H5N1)
 A/Red fox Estonia/TA2126820 21VIR10433-13/2021 (H5N1)
67
 A/chicken/France/21P013076/2021 (H5N1)
72
 A/chicken/Spain/622-8 22VIR2142-34/2022 (H5N1)
80
 A/common crane/Spain/597-2 22VIR2142-15/2022 (H5N1)
 A/Black-headed gull/Netherlands/1/2022 (H5N1)
 A/fox/Ireland/3866 22VIR2064-1/2022 (H5N1)
 A/chicken/England/000187/2022 (H5N1)
61
 A/laying hen/Moldova/68-1 22VIR638-1/2022 (H5N1)
 A/swan/Germany-BW/AI00997/2022 (H5N2)
 A/chicken/Senegal/21VIR1084-3/2021 (H5N1)
66
 A/chicken/Niger/22VIR1409-13/2022 (H5N1)
 A/western marsh harrier/Netherlands/21028606-002/2021 (H5N1)
 A/white-tailed eagle/Estonia/TA2111864-2 21VIR7512-6/2021 (H5N1)|MP
 A/chicken/England/011981/2022 (H5N1)
 A/fox/Wisconsin/22-013774-002-original/2022 (H5N1)
83
 A/whooper swan/Ireland/032444 22VIR1325-2/2021 (H5N1)
 A/chicken/Missouri/22-006639-001/2022 (H5N1)
 A/American wigeon/South Carolina/AH0195145/2021 (H5N1)
87
 A/fox/Iowa/22-014421-001-original/2022 (H5N1)
 A/chicken/Delaware/22-005260-002/2022 (H5N1)
 A/chicken/Omsk/0073/2020 (H5N8)
 A/chicken/England/043683/2020 (H5N8)
 A/mute swan/Netherlands/20017547-002/2020 (H5N1)
 A/turkey/Netherlands/21028936-001005/2021 (H5N8)
 A/seal/Germany-SH/AI05373/2021 (H5N8)
 A/seal/Germany-SH/AI05377/2021 (H5N8)
 A/Herring Gull/Netherlands/1/2020 (H5N8)
 A/chicken/Kagoshima/B3T/2021 (H5N8)
89
 A/hooded crane/Kagoshima/KU-5T/2021 (H5N8
 A/european herring gull/Netherlands/21023937-002/2021 (H5N4)
 A/duck/Guangxi/S10099/2021 (H5N8)
83
 A/chicken/Vietnam/HU14-LB11/2021 (H5N8)
 A/black swan/Beijing/1/2021 (H5N8)
64
 A/crane/Kagoshima/KU-93/2021 (H5N8)
 A/Environment/Hangzhou/E1153/2021 (H5N6)
 A/wild bird/Korea/H467/2020 (H5N8)
 A/wild duck/Korea/H331/2020 (H5N8)
 A/mallard/Georgia/DT-23072/2020 (H5N8)
 A/quail/Korea/H526/2021 (H5N1)
 A/mandarin duck/Korea/WA585/2021 (H5N1)
78
 A/chicken/Kagoshima/21A6T/2021 (H5N1)
96
 A/mute swan/Czech Republic/3777/2021 (H5N8)
 A/mute swan/Poland/MB268/2021 (H5N8)
88
 A/chicken/Bulgaria/143 19VIR3315-2/2019 (H5N8)
 A/northern pintail/Hokkaido/M13/2020 (H5N8)
66
 A/duck/Korea/H548/2020 (H5N8)
99
 A/black swan/Akita/1/2016 (H5N6)
0.010

## Slide 6
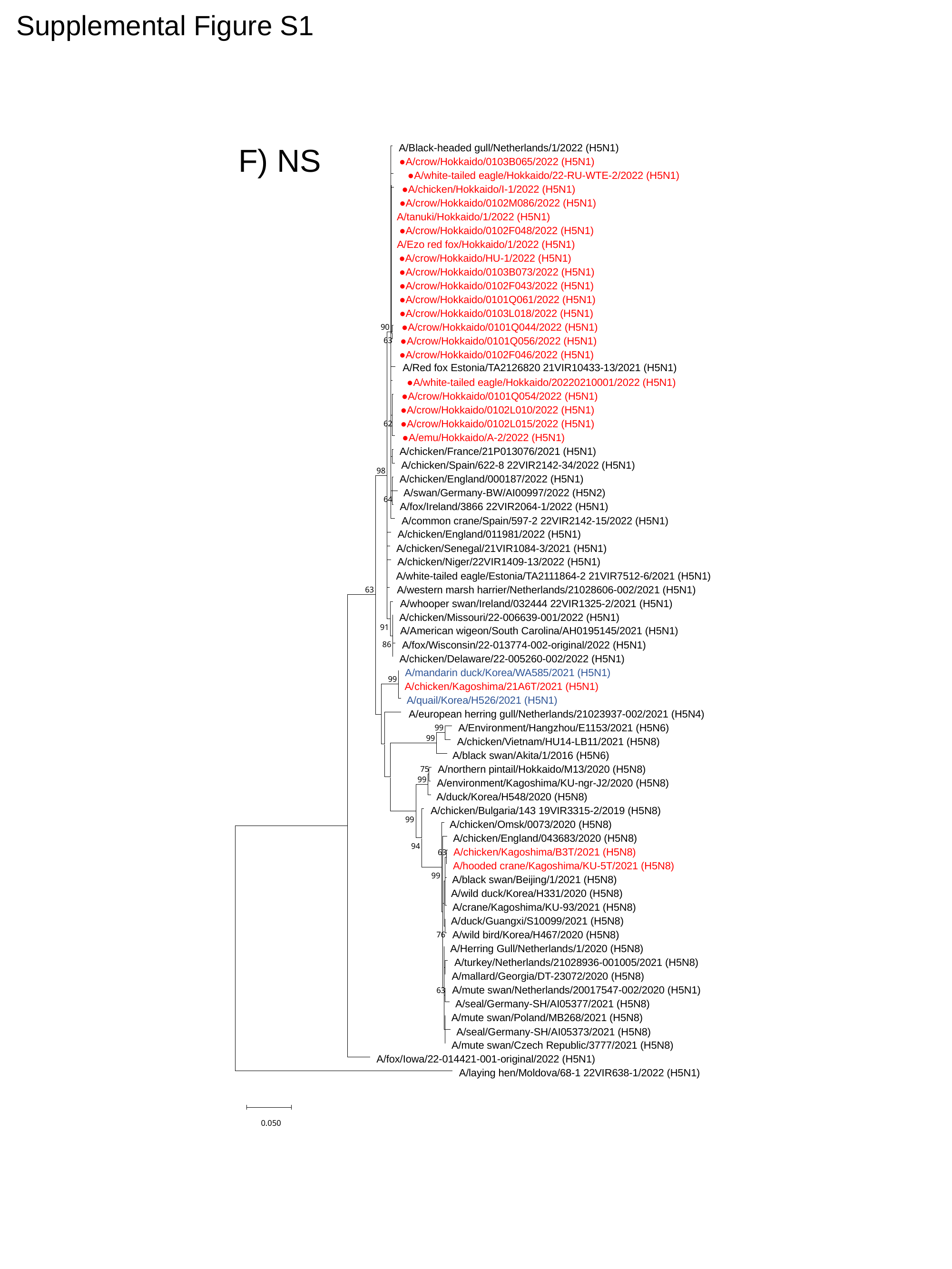

Supplemental Figure S1
F) NS
 A/Black-headed gull/Netherlands/1/2022 (H5N1)
 ●A/crow/Hokkaido/0103B065/2022 (H5N1)
 ●A/white-tailed eagle/Hokkaido/22-RU-WTE-2/2022 (H5N1)
 ●A/chicken/Hokkaido/I-1/2022 (H5N1)
 ●A/crow/Hokkaido/0102M086/2022 (H5N1)
 A/tanuki/Hokkaido/1/2022 (H5N1)
 ●A/crow/Hokkaido/0102F048/2022 (H5N1)
 A/Ezo red fox/Hokkaido/1/2022 (H5N1)
 ●A/crow/Hokkaido/HU-1/2022 (H5N1)
 ●A/crow/Hokkaido/0103B073/2022 (H5N1)
 ●A/crow/Hokkaido/0102F043/2022 (H5N1)
 ●A/crow/Hokkaido/0101Q061/2022 (H5N1)
 ●A/crow/Hokkaido/0103L018/2022 (H5N1)
 ●A/crow/Hokkaido/0101Q044/2022 (H5N1)
 ●A/crow/Hokkaido/0101Q056/2022 (H5N1)
 ●A/crow/Hokkaido/0102F046/2022 (H5N1)
 A/Red fox Estonia/TA2126820 21VIR10433-13/2021 (H5N1)
 ●A/white-tailed eagle/Hokkaido/20220210001/2022 (H5N1)
 ●A/crow/Hokkaido/0101Q054/2022 (H5N1)
 ●A/crow/Hokkaido/0102L010/2022 (H5N1)
 ●A/crow/Hokkaido/0102L015/2022 (H5N1)
 ●A/emu/Hokkaido/A-2/2022 (H5N1)
 A/chicken/France/21P013076/2021 (H5N1)
 A/chicken/Spain/622-8 22VIR2142-34/2022 (H5N1)
 A/chicken/England/000187/2022 (H5N1)
 A/swan/Germany-BW/AI00997/2022 (H5N2)
 A/fox/Ireland/3866 22VIR2064-1/2022 (H5N1)
 A/common crane/Spain/597-2 22VIR2142-15/2022 (H5N1)
 A/chicken/England/011981/2022 (H5N1)
 A/chicken/Senegal/21VIR1084-3/2021 (H5N1)
 A/chicken/Niger/22VIR1409-13/2022 (H5N1)
 A/white-tailed eagle/Estonia/TA2111864-2 21VIR7512-6/2021 (H5N1)
 A/western marsh harrier/Netherlands/21028606-002/2021 (H5N1)
63
 A/whooper swan/Ireland/032444 22VIR1325-2/2021 (H5N1)
 A/chicken/Missouri/22-006639-001/2022 (H5N1)
 A/American wigeon/South Carolina/AH0195145/2021 (H5N1)
 A/fox/Wisconsin/22-013774-002-original/2022 (H5N1)
86
 A/chicken/Delaware/22-005260-002/2022 (H5N1)
 A/mandarin duck/Korea/WA585/2021 (H5N1)
99
 A/chicken/Kagoshima/21A6T/2021 (H5N1)
 A/quail/Korea/H526/2021 (H5N1)
 A/european herring gull/Netherlands/21023937-002/2021 (H5N4)
 A/Environment/Hangzhou/E1153/2021 (H5N6)
99
99
 A/chicken/Vietnam/HU14-LB11/2021 (H5N8)
 A/black swan/Akita/1/2016 (H5N6)
 A/northern pintail/Hokkaido/M13/2020 (H5N8)
75
99
 A/environment/Kagoshima/KU-ngr-J2/2020 (H5N8)
 A/duck/Korea/H548/2020 (H5N8)
 A/chicken/Bulgaria/143 19VIR3315-2/2019 (H5N8)
99
 A/chicken/Omsk/0073/2020 (H5N8)
 A/chicken/England/043683/2020 (H5N8)
94
 A/chicken/Kagoshima/B3T/2021 (H5N8)
63
 A/hooded crane/Kagoshima/KU-5T/2021 (H5N8)
99
 A/black swan/Beijing/1/2021 (H5N8)
 A/wild duck/Korea/H331/2020 (H5N8)
 A/crane/Kagoshima/KU-93/2021 (H5N8)
 A/duck/Guangxi/S10099/2021 (H5N8)
 A/wild bird/Korea/H467/2020 (H5N8)
76
 A/Herring Gull/Netherlands/1/2020 (H5N8)
 A/turkey/Netherlands/21028936-001005/2021 (H5N8)
 A/mallard/Georgia/DT-23072/2020 (H5N8)
 A/mute swan/Netherlands/20017547-002/2020 (H5N1)
63
 A/seal/Germany-SH/AI05377/2021 (H5N8)
 A/mute swan/Poland/MB268/2021 (H5N8)
 A/seal/Germany-SH/AI05373/2021 (H5N8)
 A/mute swan/Czech Republic/3777/2021 (H5N8)
 A/fox/Iowa/22-014421-001-original/2022 (H5N1)
 A/laying hen/Moldova/68-1 22VIR638-1/2022 (H5N1)
90
63
62
98
64
91
0.050

## Slide 7
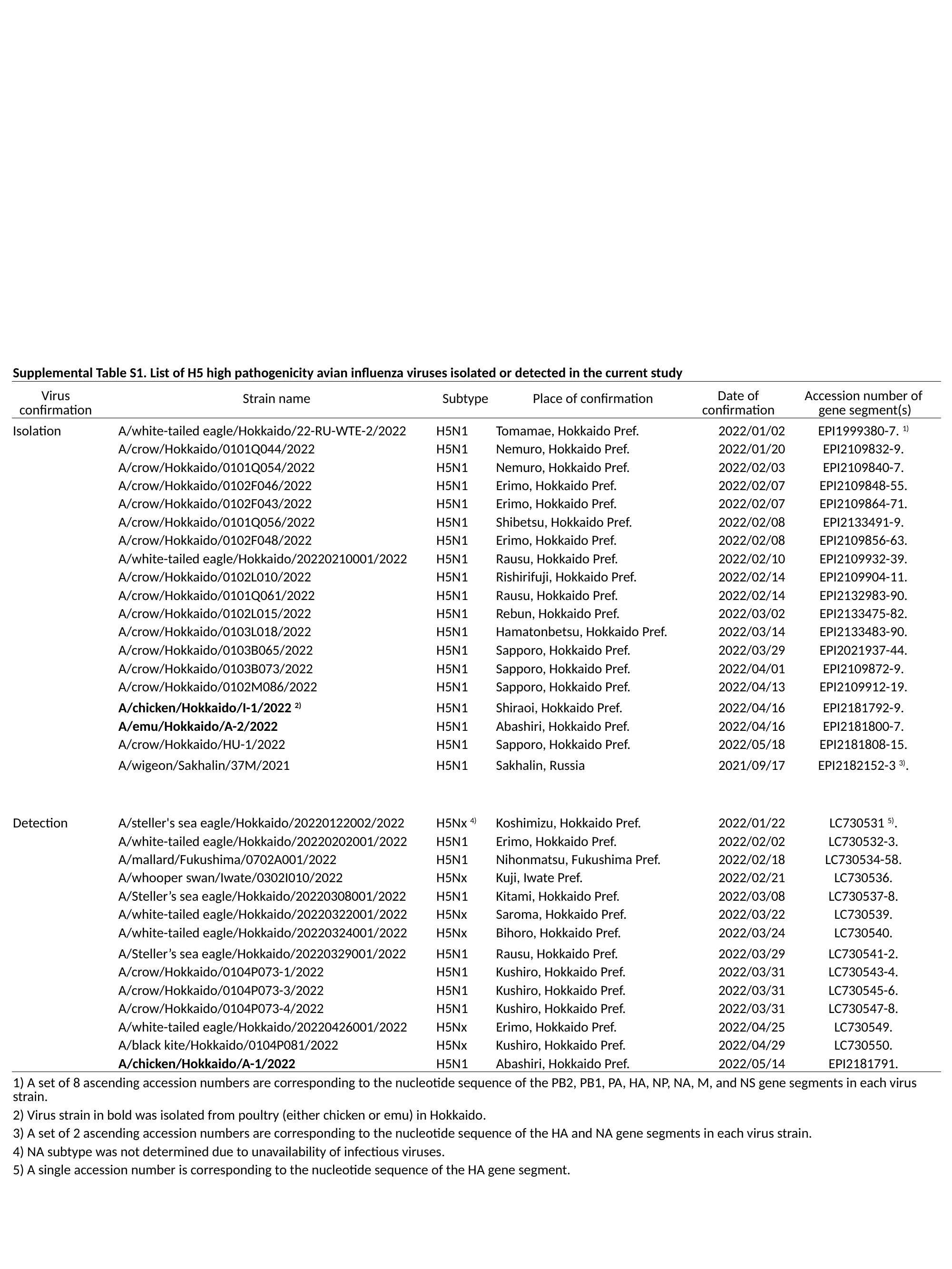

| Supplemental Table S1. List of H5 high pathogenicity avian influenza viruses isolated or detected in the current study | | | | | | |
| --- | --- | --- | --- | --- | --- | --- |
| Virus confirmation | | Strain name | Subtype | Place of confirmation | Date of confirmation | Accession number of gene segment(s) |
| Isolation | | A/white-tailed eagle/Hokkaido/22-RU-WTE-2/2022 | H5N1 | Tomamae, Hokkaido Pref. | 2022/01/02 | EPI1999380-7. 1) |
| | | A/crow/Hokkaido/0101Q044/2022 | H5N1 | Nemuro, Hokkaido Pref. | 2022/01/20 | EPI2109832-9. |
| | | A/crow/Hokkaido/0101Q054/2022 | H5N1 | Nemuro, Hokkaido Pref. | 2022/02/03 | EPI2109840-7. |
| | | A/crow/Hokkaido/0102F046/2022 | H5N1 | Erimo, Hokkaido Pref. | 2022/02/07 | EPI2109848-55. |
| | | A/crow/Hokkaido/0102F043/2022 | H5N1 | Erimo, Hokkaido Pref. | 2022/02/07 | EPI2109864-71. |
| | | A/crow/Hokkaido/0101Q056/2022 | H5N1 | Shibetsu, Hokkaido Pref. | 2022/02/08 | EPI2133491-9. |
| | | A/crow/Hokkaido/0102F048/2022 | H5N1 | Erimo, Hokkaido Pref. | 2022/02/08 | EPI2109856-63. |
| | | A/white-tailed eagle/Hokkaido/20220210001/2022 | H5N1 | Rausu, Hokkaido Pref. | 2022/02/10 | EPI2109932-39. |
| | | A/crow/Hokkaido/0102L010/2022 | H5N1 | Rishirifuji, Hokkaido Pref. | 2022/02/14 | EPI2109904-11. |
| | | A/crow/Hokkaido/0101Q061/2022 | H5N1 | Rausu, Hokkaido Pref. | 2022/02/14 | EPI2132983-90. |
| | | A/crow/Hokkaido/0102L015/2022 | H5N1 | Rebun, Hokkaido Pref. | 2022/03/02 | EPI2133475-82. |
| | | A/crow/Hokkaido/0103L018/2022 | H5N1 | Hamatonbetsu, Hokkaido Pref. | 2022/03/14 | EPI2133483-90. |
| | | A/crow/Hokkaido/0103B065/2022 | H5N1 | Sapporo, Hokkaido Pref. | 2022/03/29 | EPI2021937-44. |
| | | A/crow/Hokkaido/0103B073/2022 | H5N1 | Sapporo, Hokkaido Pref. | 2022/04/01 | EPI2109872-9. |
| | | A/crow/Hokkaido/0102M086/2022 | H5N1 | Sapporo, Hokkaido Pref. | 2022/04/13 | EPI2109912-19. |
| | | A/chicken/Hokkaido/I-1/2022 2) | H5N1 | Shiraoi, Hokkaido Pref. | 2022/04/16 | EPI2181792-9. |
| | | A/emu/Hokkaido/A-2/2022 | H5N1 | Abashiri, Hokkaido Pref. | 2022/04/16 | EPI2181800-7. |
| | | A/crow/Hokkaido/HU-1/2022 | H5N1 | Sapporo, Hokkaido Pref. | 2022/05/18 | EPI2181808-15. |
| | | A/wigeon/Sakhalin/37M/2021 | H5N1 | Sakhalin, Russia | 2021/09/17 | EPI2182152-3 3). |
| | | | | | | |
| | | | | | | |
| Detection | | A/steller's sea eagle/Hokkaido/20220122002/2022 | H5Nx 4) | Koshimizu, Hokkaido Pref. | 2022/01/22 | LC730531 5). |
| | | A/white-tailed eagle/Hokkaido/20220202001/2022 | H5N1 | Erimo, Hokkaido Pref. | 2022/02/02 | LC730532-3. |
| | | A/mallard/Fukushima/0702A001/2022 | H5N1 | Nihonmatsu, Fukushima Pref. | 2022/02/18 | LC730534-58. |
| | | A/whooper swan/Iwate/0302I010/2022 | H5Nx | Kuji, Iwate Pref. | 2022/02/21 | LC730536. |
| | | A/Steller’s sea eagle/Hokkaido/20220308001/2022 | H5N1 | Kitami, Hokkaido Pref. | 2022/03/08 | LC730537-8. |
| | | A/white-tailed eagle/Hokkaido/20220322001/2022 | H5Nx | Saroma, Hokkaido Pref. | 2022/03/22 | LC730539. |
| | | A/white-tailed eagle/Hokkaido/20220324001/2022 | H5Nx | Bihoro, Hokkaido Pref. | 2022/03/24 | LC730540. |
| | | A/Steller’s sea eagle/Hokkaido/20220329001/2022 | H5N1 | Rausu, Hokkaido Pref. | 2022/03/29 | LC730541-2. |
| | | A/crow/Hokkaido/0104P073-1/2022 | H5N1 | Kushiro, Hokkaido Pref. | 2022/03/31 | LC730543-4. |
| | | A/crow/Hokkaido/0104P073-3/2022 | H5N1 | Kushiro, Hokkaido Pref. | 2022/03/31 | LC730545-6. |
| | | A/crow/Hokkaido/0104P073-4/2022 | H5N1 | Kushiro, Hokkaido Pref. | 2022/03/31 | LC730547-8. |
| | | A/white-tailed eagle/Hokkaido/20220426001/2022 | H5Nx | Erimo, Hokkaido Pref. | 2022/04/25 | LC730549. |
| | | A/black kite/Hokkaido/0104P081/2022 | H5Nx | Kushiro, Hokkaido Pref. | 2022/04/29 | LC730550. |
| | | A/chicken/Hokkaido/A-1/2022 | H5N1 | Abashiri, Hokkaido Pref. | 2022/05/14 | EPI2181791. |
| 1) A set of 8 ascending accession numbers are corresponding to the nucleotide sequence of the PB2, PB1, PA, HA, NP, NA, M, and NS gene segments in each virus strain. | | | | | | |
| 2) Virus strain in bold was isolated from poultry (either chicken or emu) in Hokkaido. | | | | | | |
| 3) A set of 2 ascending accession numbers are corresponding to the nucleotide sequence of the HA and NA gene segments in each virus strain. | | | | | | |
| 4) NA subtype was not determined due to unavailability of infectious viruses. | | | | | | |
| 5) A single accession number is corresponding to the nucleotide sequence of the HA gene segment. | | | | | | |
